# Slight Disruption in Intestinal Environment by Dextran Sodium Sulfate Reduces Egg Yolk Size Through Disfunction of Ovarian Follicle Growth

**DOI:** 10.3389/fphys.2020.607369

**Published:** 2021-01-15

**Authors:** Takahiro Nii, Takashi Bungo, Naoki Isobe, Yukinori Yoshimura

**Affiliations:** ^1^Graduate School of Integrated Sciences for Life, Hiroshima University, Higashi-Hiroshima, Japan; ^2^Research Center for Animal Science, Hiroshima University, Higashi-Hiroshima, Japan

**Keywords:** chicken, intestinal environment, egg yolk, dextran sodium sulfate, very-low-density lipoprotein

## Abstract

Intestinal environments such as microbiota, mucosal barrier function, and cytokine production affect egg production in laying hens. Dextran sodium sulfate (DSS) is an agent that disrupts the intestinal environment. Previously, we reported that the oral administration of dextran sodium sulfate (DSS: 0.9 g/kg BW) for 5 days caused severe intestinal inflammation in laying hens. However, the DSS concentration in the previous study was much higher to induce a milder disruption of the intestinal environment without heavy symptoms. Thus, the goal of this study was to determine the effects of a lower dose of DSS on the intestinal environment and egg production in laying hens. White Leghorn laying hens (330-day old) were oral administered with or without 0.225 g DSS/kg BW for 28 days (DSS and control group: *n* = 7 and 8, respectively). Weekly we collected all laid eggs and blood plasma samples. Intestinal tissues, liver, ovarian follicles, and the anterior pituitary gland were collected 1 day after the final treatment. Lower concentrations of orally administered DSS caused (1) a decrease in the ratio of villus height/crypt depth, *occludin* gene expressions in large intestine and cecal microbiota diversity, (2) a decrease in egg yolk weight, (3) an increase in VLDLy in blood plasma, (4), and enhanced the egg yolk precursor accumulation in the gene expression pattern in the follicular granulosa layer, (5) an increase in *FSH* and *IL-1*β gene expression in the pituitary gland, and (6) an increase in concentration of plasma lipopolysaccharide binding protein. These results suggested that the administration of the lower concentration of DSS caused a slight disruption in the intestinal environment. This disruption included poor intestinal morphology and decreased cecal microbiome diversity. The change in the intestinal environment decreases egg yolk size without decreasing the VLDLy supply from the liver. The decrease in egg yolk size is likely to be caused by the dysfunction of egg-yolk precursor uptake in ovarian follicles. In conclusion, the oral administration of a lower dose of DSS is an useful method to cause slight disruptions of intestinal environment, and the intestinal condition decreases egg yolk size through disfunction of ovarian follicle.

## Introduction

The intestine is the primary organ for digestion and absorption of nutrients. The healthy maintenance of the intestine is important for sustainable meats and egg production in chickens. Intestinal health is closely related to the condition of the intestinal environment, such as the balance of microbiota, mucosal barrier function, and cytokine production. A good intestinal environment contributes to the efficiency of feed digestion and nutrient absorptive function, preventing microbial infection and enhancing chicken egg and meat production (Possemiers et al., 2009; Shang et al., 2018). Therefore, the control of the intestinal environment is important for best practice poultry production.

Intestinal infection by pathogen such as *Clostridium* and *Eimeria* disrupts the intestinal environment, namely the induction of pro-inflammatory cytokine production, such as interleukin (IL)-1β and IL-6, and histological inflammatory events in the intestinal mucosa (Hong et al., 2006; Belote et al., 2018; Fasina and Lillehoj, 2019). It has been reported that *C. perfringes* and *E. tenella* infection also cause changes in cecal microflora composition in broiler chickens (Stanley et al., 2014; Lin et al., 2017). In addition, pathogenic infections in the intestine of layer and broiler breeder hens reduce their egg and meat production (Klasing, 2007; Lensing et al., 2012; Ritzi et al., 2014). Accordingly, it is suggested that pathogen infection causes disruption in the intestinal environment, including intestinal inflammation and a decline in poultry productivity. However, the mechanism by which egg production is reduced remains unknown. Furthermore, the disruption of the intestinal environment is related to dysbiosis, intestinal barrier function, and intestinal inflammation and has become a common problem since the ban on growth-promoting antibiotics in animal feed (Huyghebaert et al., 2011; Ducatelle et al., 2018). Therefore, understanding the mechanism by which the intestinal environment affects poultry production is highly important.

Dextran sodium sulfate (DSS) is commonly used as an intestinal inflammation inducer. DSS-induced intestinal inflammation in rodents has been used in animal models of human colitis, such as inflammatory bowel disease and colitis-associated cancer (Saleh and Trinchieri, 2011; Heijmans et al., 2014). The mechanism suggested by DSS induced intestinal inflammation is as follows: orally treated DSS directly injures intestinal epithelial cells, enteric bacteria translocate into the lamina propria from the lumen through the lesion, and the translocated bacteria and DSS in the mucosal tissue attract and activate immune cells, resulting in intestinal inflammation (Saleh and Trinchieri, 2011). Mice administered DSS in drinking water showed a higher serum IL-6 concentration and histological intestinal inflammatory events as well as profound changes in colon microflora (Hakansson et al., 2015). Therefore, we suggest that DSS is suitable to use to disrupt the intestinal environment. Recently, the DSS caused intestinal inflammation method had started to be used in chicks for poultry intestinal research (Kuttappan et al., 2015, 2016; Menconi et al., 2015; Simon et al., 2016). We previously reported that the oral administration of DSS (0.9 g/kg BW) for 5 days caused severe intestinal inflammation and reduced egg laying in laying hens through the disruption of the egg yolk precursor production in association with liver inflammation (Nii et al., 2020). This experiment mimics the acute intestinal inflammation caused by pathogenic bacterial infection. DSS caused severe inflammation in the intestinal mucosa with inflammatory symptoms such as bloody stools, anorexia, and body weight loss. Thus, the DSS treatment condition in our previous study was suitable for the mediation of severe acute intestinal inflammation, but the condition was too strong to induce a milder disruption of the intestinal environment without heavy symptoms. Simon et al. (2016) reported a treatment with 2.5% DSS in drinking water caused histological intestinal damage in the colon, but not when administering a lower concentration of DSS. We hypothesize that an oral administration of a lower concentration of DSS when compared with our previous study (0.9 g/kg BW), causes a milder disruption of the intestinal environment without heavy symptoms such as bloody stools, anorexia, and body weight loss.

The goal of this study was to determine the effects of the oral administration of a lower dose of DSS on the intestinal environment and egg production in laying hens. We focused on whether (1) lower concentration DSS disrupted the intestinal environment, (2) the DSS mediated disruption of the intestinal environment affects egg production, (3) egg yolk precursor production in the liver is inhibited, (4) the accumulation of egg yolk precursor into ovarian follicles is affected, and (5) the endocrine regulation related egg production in the pituitary gland is changed.

## Materials and Methods

### Experimental Birds

White Leghorn hens regularly laying seven or more eggs in a clutch (~330-days old) were housed separating in individual wire cages (W300 × D400 × H600 mm) equipped individual feed cup and water cup with a 14:10-h light-dark cycle. They were provided feed and water *ad libitum*. The birds were divided into two groups, namely the control (CON) and DSS groups (*n* = 8 and 7, respectively). Birds were administered a single oral dose of sterilized water with or without 0.225 g DSS/kg body weight (molecular weight: 5,000–6,000, Nacalai Tesque, Inc., Kyoto, Japan) by cannula for 28 days (DSS and CON groups, respectively).

The total number of eggs laid during the experimental period (28 days) was recorded. The egg-laying ratio was calculated for each hen group. Laid eggs were collected once a week, and the whole egg, egg yolk, and eggshell weights were measured. Blood was collected once a week at 11:00–12:00 (~2–3 h after oviposition). The blood samples were inverted with heparin (Mochida Pharmaceutical Co., Ltd., Tokyo, Japan). Plasma samples were separated by centrifugation (1,000 × g, 10 min, room temperature), and stored at −80°C until used for the biochemical analysis of TG, total cholesterol (T-CHO), and enzyme-linked immunosorbent assay (ELISA) analysis of lipopolysaccharide binding protein (LBP) concentrations.

Birds were euthanized under anesthesia with sodium pentobarbital (Somnopentyl; Kyoritsu Seiyaku Corporation, Tokyo, Japan), and the intestinal tissues [jejunum, ileum, cecum, and rectum (we collected one to three centimeter of lower part tissues from ileum-cecum junction, and defined that part as rectum)], liver, follicular granulosa layer (F1 and F5 follicles), and anterior pituitary gland were collected. Intestine and liver tissue samples were processed into paraffin sections. All tissue samples were used for total RNA extraction in same day. In addition, cecal contents were also collected (*n* = 4, each group) for microbiota analysis.

The experiment was done three independent experimental repetitions, namely 1st: CON (*n* = 2) and DSS (*n* = 2), 2nd: CON (*n* = 2) and DSS (*n* = 2), 3rd: CON (*n* = 4) and DSS (*n* = 3), and we pooled all samples for each analysis. All experiments in this study were approved by the Hiroshima University Animal Research Committee (Approval No. C17-3). We adhered to all animal handling regulations.

### Tissue Preparation and Staining for Histology

Intestinal and liver tissues were fixed with 10% (v/v) formalin in PBS and processed into paraffin sections (4 μm thick). Sections were stained with Hansen's hematoxylin and eosin for histological observation. Intestinal tissue sections were examined under a light microscope connected to image analysis software (NIS-Elements; Nikon, Tokyo, Japan). The ratio of the villus height/crypt depth and the height of epithelial cells of the jejunum, ileum, cecum, and rectum were measured. The measurement was performed in triplicate on a section from each sample, and the average was calculated.

### Real-Time Polymerase Chain Reaction (PCR) Analysis for the Expression of Inflammation and Lipid Metabolism-Related Genes

Total RNA was extracted from the liver, follicular granulosa layer, and anterior pituitary gland using Sepasol RNA I Super (Nacalai Tesque, Inc.). Total RNA was extracted and purified from intestinal mucosal samples (jejunum, ileum, cecum, and rectum) using the NucleoSpin RNA kit (Macherey-Nagel GmbH & Co. KG., Duren, Germany), which is a column-type RNA extraction kit because any remaining DSS in the intestinal tissues would inhibit the PCR (Kerr et al., 2012). The extracted total RNA samples were dissolved in TE buffer (10 mM Tris-HCl, pH 8.0, with 1 mM EDTA) and stored at −80°C until RT-PCR. The concentration of total RNA in each sample was measured using a Nano Drop Lite (Thermo Fisher Scientific, MA, USA). The RNA samples were reverse transcribed using ReverTra Ace qPCR RT Master Mix with gDNA Remover (Toyobo Co., Ltd., Osaka, Japan) on a PTC-100 programmable thermal controller (MJ Research, Waltham, MA, USA), programmed according to the manufacturer's instructions. Real-time PCR was performed using an AriaMX real-time PCR system (Agilent Technologies, CA, USA) with Brilliant III Ultra-Fast SYBR Green QPCR Master Mix (Agilent Technologies). Table 1 shows the primers used for the PCR. The cycle parameters used for amplification were as follows: denaturation at 95°C for 5 s and annealing at 58°C (for *TGF*β*-2* and *TGF*β*-4*), 60°C (for *IL-6, ApoB, Claudin-1, LDLr, LR8, FSH, LH*, and *RPS17*), 62°C [for *very low-density lipoprotein (VLDL)-II, vitellogenin* (*VTG*)*-II* and *Claudin5*], or 63°C (for *IL-1*β) for 10 s, and the denaturation and annealing steps were performed for 50 cycles. The cycle parameters for the melting step were 95°C for 30 s, 65°C for 30 s, and 95°C for 30 s. RNA expression levels were calculated by the relative quantification method using a standard curve generated with serially diluted PCR products of each target gene. The relative expression level of the target mRNA in each sample was normalized to the RPS17 housekeeping gene, and was described as the mean fold change when compared to a standard sample from the control group.

**Table 1:** PCR primers used for mRNA expression analysis.

**Target genes**	**Forward primer**	**Revers primer**	**Product size**	**Accession no**.
*IL-1β*	GTGAGGCTCAACATTGCGCTGTA	TGTCCAGGCGGTAGAAGATGAAG	214	NM_204524.1
*IL-6*	AGAAATCCCTCCTCGCCAAT	AAATAGCGAACGGCCCTCA	121	NM_204628.1
*TGFβ-2*	AGGAATGTGCAGGATAATT	ATTTTGGGTGTTTTGCCAA	269	NM_001031045.3
*TGFβ-4*	ATGAGTATTGGGCCAAAG	ACGTTGAACACGAAGAAG	109	NM_001318456.1
*ApoB*	CTGCAAATGCTGGGCTGTTT	CTGGTTGAGCCATCCAGCTT	106	NM_001044633.1
*VLDL-II*	AGGGCTGAACTGGTACCAACAAAC	GGATGACCAGCCAGTCACGA	140	NM_205483.2
*VTG-II*	CAACATATCTTCCGCTTGTAACATTG	TTCACAACAAAGATTTCTCCAGTAGC	147	NM_001031276
*Claudin1*	GACTCGCTGCTTAAGCTGGA	AAATCTGGTGTTAACGGGTG	276	NM_001013611.2
*Claudin5*	GTCCCGCTCTGCTGGTTC	CCCTATCTCCCGCTTCTGG	84	NM_204201.1
*LDLr*	GGAGCAGTCACAGCATCAGCT	CTGTGTCACACTCCGCTGTCTC	109	NM_204452.1
*LR8*	TGCTACAGGAGTGTGCAAGG	TCTCTCAAGGCCAATCTTCC	88	NM_205229.1
*FSH*	TGCTTCACAAGGGATCCAG	CAGCCAGGGATCTTCACTGT	104	NM_204257.1
*LH*	GTGTCGCCCCATAAACGTAA	CAAAGGGCTGCGATACACC	124	HQ872606.1
*GnRHr*	ACCTCGGCCACTCACTGC	CACCTTTGCTGCTTTTGTGAAC	63	NM_204653.1
*RPS17*	AAGCTGCAGGAGGAGGAGAGG	GGTTGGACAGGCTGCCGAAGT	136	NM_204217.1

### Microbiome Analysis

The cecal contents at day 28 were collected and stored at −80°C until use (*n* = 4, each group). DNA from these cecal contents was extracted using the NucleoSpin DNA Stool (Macherey-Nagel GmbH & Co., Dürer, Germany) according to the manufacturer's instructions. 2-step tailed PCR was performed to process the amplicon of the V3 and V4 regions of the 16S rRNA genes. Sequencing was conducted using the Miseq system (Illumina, CA, USA) for 300 × 2 bp. Two-step tailed PCR and sequencing were performed by Bioengineering Lab Co., Ltd (Kanagawa, Japan).

The sequenced fast-q data were analyzed using QIIME2 (ver. 2020.2) with Greengenes 13_8 99% of OTUs full-length sequences for reference. The first 22 bases of all reads were trimmed to achieve a length of 250 bases and they were denoised by dada2. The relative abundance of taxa at the family level of the groups was presented as a mean % value. The alpha diversity, as species richness, was measured using the observed OTUs. Beta diversity plots, which suggest changes in species diversity among samples, were constructed to visualize the distance of the samples using unweighted UniFrac.

### Biochemical Assays of Blood Plasma

Blood parameters, namely TG and T-CHO, were measured by a Beckman Colter AU480 automatic biochemistry analysis system (Beckman Coulter, Inc., CA, USA) using reagent kits provided by the manufacturer.

The VLDLy (particle size with diameter of 25–44 nm) was separated, and cholesterol (CHO) and TG concentrations contained in the VLDLy fraction were measured by high performance liquid chromatography (HPLC) using LipoSEARCH service in Funakoshi Co., Ltd (*n* = 4, each group).

### ELISA Protocol

Plasma corticosterone and estradiol-17β were analyzed by a competitive enzyme immunoassay as described previously (Isobe and Nakao, 2003). The lipid phase of the blood plasma was extracted with dichloromethane. The microplate wells were precoated with anti-rabbit IgG antibody (Sato et al., 2011) for 4 h. Then we added anti-corticosterone antibody (Cosmo Bio Co., Ltd., Tokyo, Japan) or anti-estradiol-17β antibody (Cosmo Bio Co., Ltd.), and HRP-conjugated corticosterone (FKA 419; Cosmo Bio Co., Ltd) or HRP-conjugated estradiol-17β (FKA 236-E; Cosmo Bio Co., Ltd) for 4 h at 20°C. The plates were washed three times with phosphate-buffered saline (PBS) containing Tween 20, and then 3,3′,5,5′-tetramethylbenzidine substrate solution was added, and the plate was incubated for 30 min. The optical density was read at 450 nm using a microplate reader (Multiskan FC; Thermo Fisher Scientific). The corticosterone and estradiol-17β concentrations were calculated using the absolute quantification method with a standard curve.

The plasma concentration of chicken LBP was analyzed with a sandwich-ELISA principle using a commercially available kit (Chicken LBP ELISA Kit; Catalog No: MBS2503399, MyBioSource, Inc., CA, USA). Plasma samples were centrifuged (15,000 × rpm, 60 min, 4°C) and the top lipid-rich layer was removed before ELISA. The intermediate translucent layer was used for ELISA because the samples with high lipid numbers were not suitable for ELISA assay.

### Statistical Analysis

Values are expressed as mean ± SEM. The significant differences between the CON and DSS groups in the ratio of villus heights/crypt depth, mRNA expression levels, and CHO and TG concentrations in VLDLy in each tissue were evaluated by Student's *t*-test for homoscedastic samples and Welch's *t*-test for heteroscedastic samples. Egg parameters and all blood parameters in the control and DSS groups on days 0, 7, 14, 21, and 28 were analyzed by two-way repeated measures ANOVA and Tukey's test to assess the significance of the interaction between time and DSS treatment. The significant difference in OTU numbers between CON and DSS groups on alpha-diversity was calculated by Kruskal-Wallis analysis. The pairwise PERMANOVA analysis method in QIIME2 was performed on the unweighted UniFrac distance matrix of 8 samples for beta-diversity. The significance of the PERMANOVA was obtained by a 999-permutation test. Differences were considered significant when the *P*-value was <0.05.

## Results

### Body Weight and Feed Intake

Average body weight in the CON group was 1.66, 1.64, 1.62, 1.63, and 1.62 kg on day 0, 7, 14, 21, 28. Average body weight in the DSS group was 1.66, 164, 1.64, 1.64, and 1.63 kg on day 0, 7, 14, 21, 28, respectively. The average feed intake per day in the CON group was 88.4, 94.3, 95.3, 103.1, and 96.3 g on day 0, 7, 14, 21, 28. The average feed intake per day in the DSS group was 94.9, 91.1, 97.4, 97.0, and 96.6 g on day 0, 7, 14, 21, 28, respectively. The body weight and feed intake results between the CON and DSS groups were not significantly different throughout the experiment.

### Intestinal Structure, Mucosal Cytokine Expression, and Cecal Microflora

**Figure 1:**
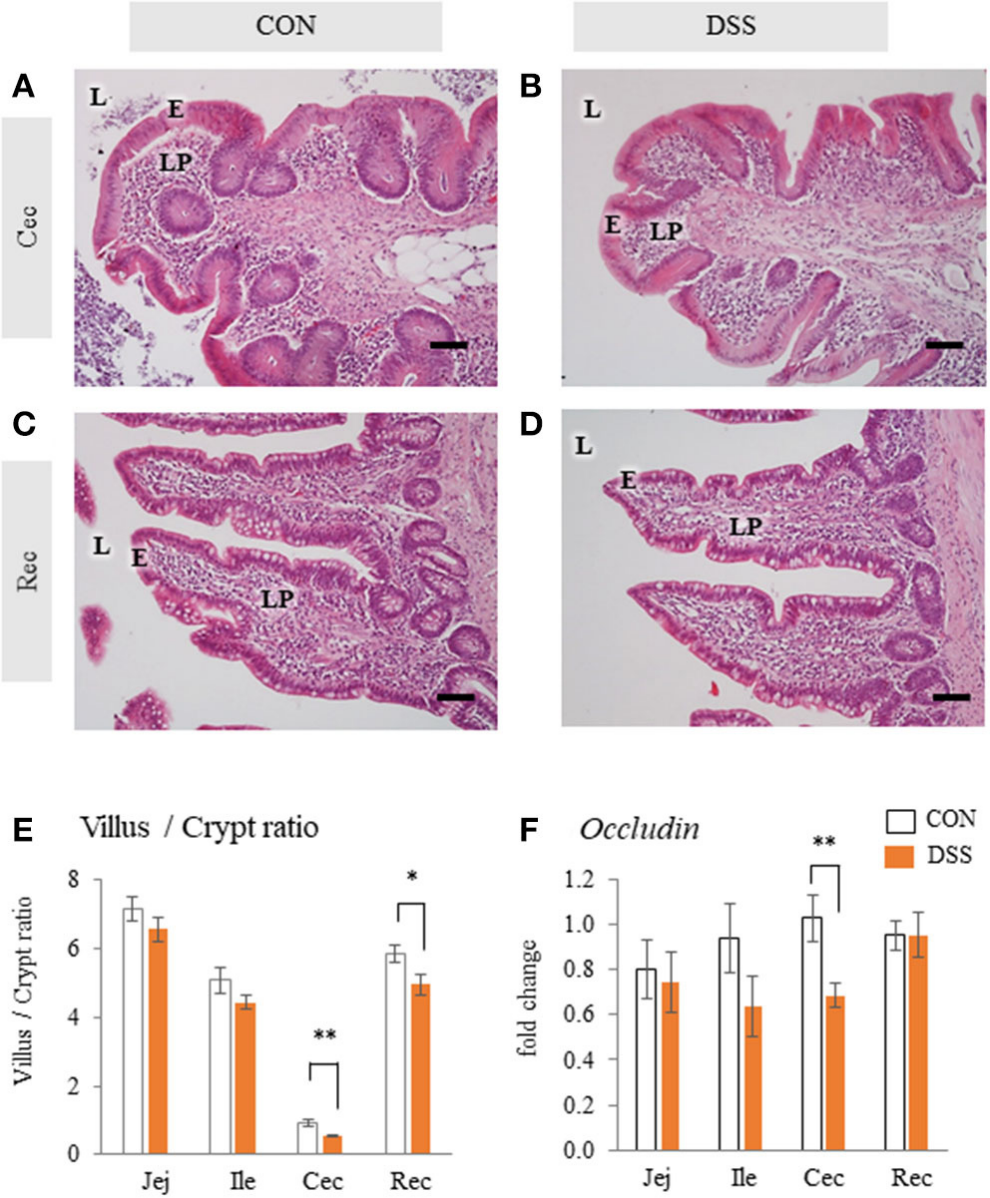
Histology and *Occludin* mRNA expression in the intestinal mucosa with or without dextran sodium sulfate (DSS). Micrographs demonstrate the HE staining of the cecum [Cec: **(A)** and **(B)**] and rectum [Rec: **(C)** and **(D)**] in hens orally administered DSS (DSS group) or water (CON group). E = mucosal epithelium, L = lumen, LP = lamina propria. Scale bars = 50 μm. **(E)** Ratio of villus height/ crypt depth of jejunum (Jej), ileum (Ile), Cec, and Rec. Open bars represent the control group, and orange filled bars represent the DSS group. Values are the ratio of villus height/crypt depth (CON and DSS, *n* = 8 and 7). **(F)** Effects of the oral administration of DSS on the mRNA expression of *Occludin* in the intestinal mucosa. Values are the fold change in target gene expression when compared to a standard sample from the CON group of each segment (CON and DSS, *n* = 8 and 7). Target gene expression was normalized to the house-keeping gene *RPS17*. Asterisks (*, **) indicate significant differences between the CON and DSS groups (*P* < 0.05 and *P* < 0.01, respectively).

The mucosal tissue of the cecum did not show any histological damage, such as disintegration of epithelial cells and a large amount of red blood cell accumulation in the lamina propria in either the CON or the DSS groups (Figures 1A,B). The rectum did not show any histological damage in either the CON or DSS groups (Figures 1C,D). However, the ratios of villus height/crypt depth in the cecum and rectum were significantly lower in the DSS group than in the CON group, but not in the jejunum and ileum (Figure 1E). In addition, the expression of *occludin* (a mucosal protein content of tight junctions) was lower in the DSS group than in the CON group in the cecum (Figure 1F).

**Figure 2:**
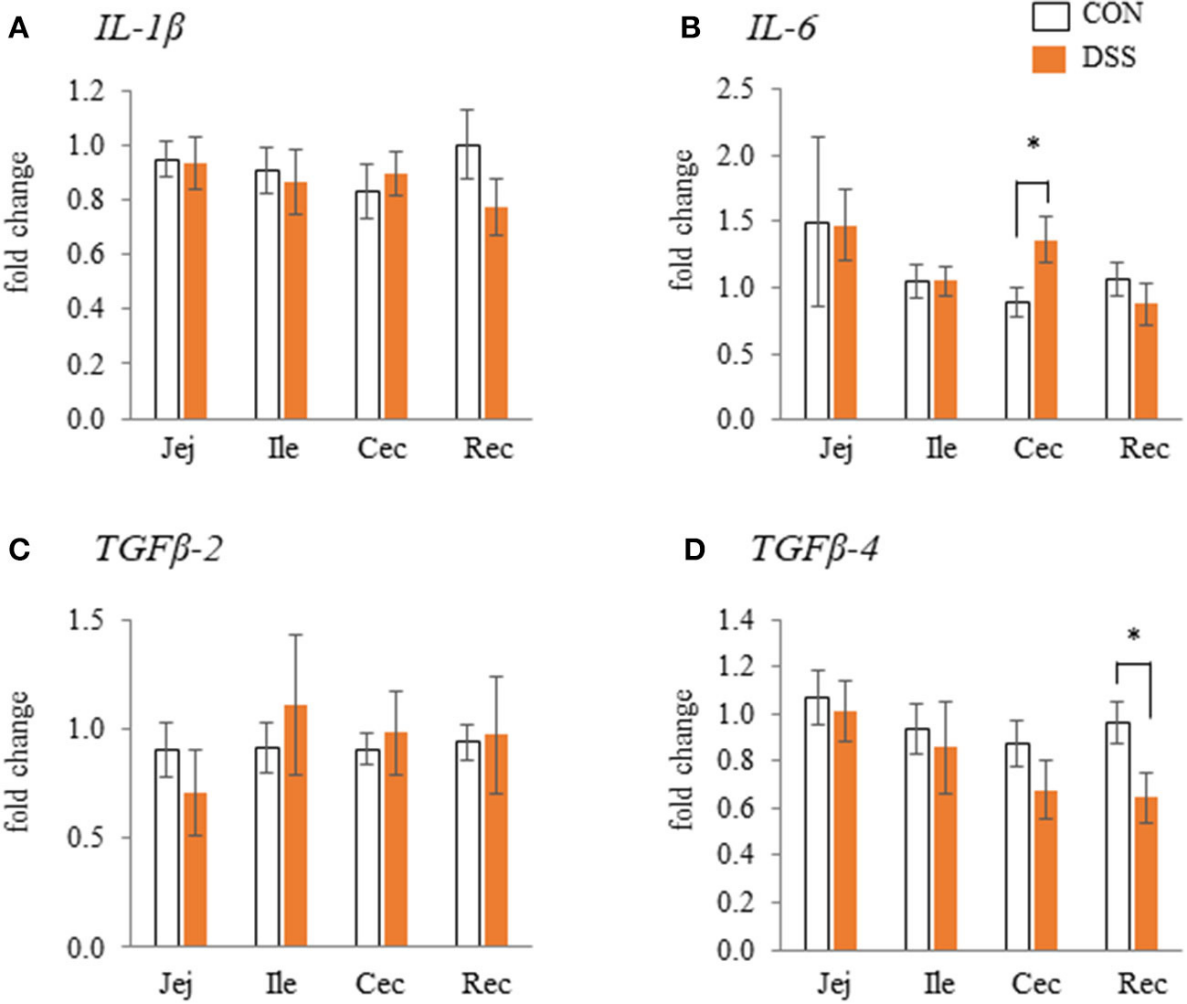
The effects of dextran sodium sulphate (DSS) on the mRNA expression of pro- [IL-1β and IL-6: **(A)** and **(B)**] and anti- [TGFβ-2 and 4: **(C)** and **(D)**] inflammatory cytokines in the mucosal tissues of the jejunum (Jej), ileum (Ile), cecum (Cec), and rectum (Rec). The effects of dextran sodium sulfate (DSS) on the mRNA expression of pro- and anti-inflammatory cytokines in the mucosal tissues of the jejunum (Jej), ileum (Ile), cecum (Cec), and rectum (Rec). Open bars represent the CON group, and orange filled bars represent the DSS group. Values are the fold change in the target gene expression when compared to a standard sample from the CON group of each segment (CON and DSS, *n* = 8 and 7). The target gene expression was normalized to the house-keeping gene *RPS17*. Asterisks (*) indicate significant differences between the water (CON) and DSS-treated groups (*P* < 0.05).

The gene expression levels of *IL-6* in the cecum were significantly higher in the DSS group than in the CON group, but not in the jejunum, ileum, and rectum (Figure 2B). The gene expression level of *TGF-*β*4* in the rectum was significantly lower in the DSS group than in the control group (Figure 2D). However, no significant differences in expression were observed in the other segments. The *IL-1*β and *TGF*β*-2* gene expression levels in all intestinal segments were not significantly different between the CON and DSS groups (Figures 2A,C).

**Figure 3:**
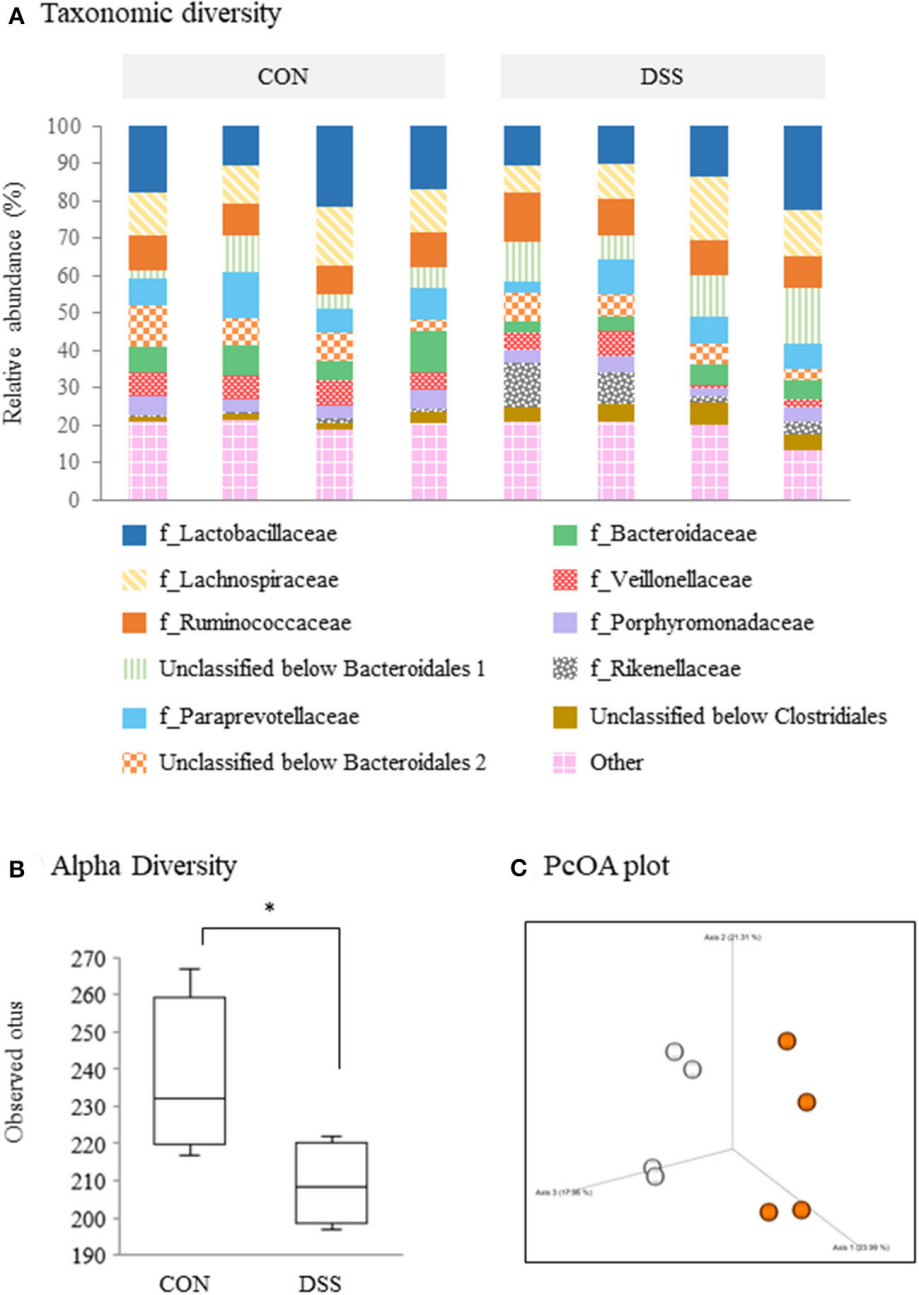
Microbiome profiles in the cecum contents of laying hens administered dextran sodium sulfate (DSS group) or water (CON group). **(A)** A taxonomic diversity bar plot showing relative abundance of taxa identified to the family level (*n* = 4). **(B)** Alpha-diversity boxplot of observed OTUs. Boxplots show the quartiles, median, and extremities of the value. Asterisk (*) indicate significant differences between the water (CON) and DSS-treated groups (*P* < 0.05). **(C)** 3D PCoA plot based on an unweighted UniFrac distance matrix. White circles represent the CON group, and orange circles represent the DSS group.

Figure 3 shows the microbiome analysis of the cecal contents using eight samples (four randomly selected samples from the CON and DSS groups). A taxonomic bar plot (to the family level) is shown in Figure 3A. *Lactobacillaceae, Lachnospiraceae*, and *Ruminococcaceae* were in higher frequencies in all hens. The frequencies decreased in order from unclassified *Bacteroidales* 1, *Paraprevotellaceae*, unclassified *Bacteroidales* 2, *Bacteroidaceae, Veillonellaceae, Porphyromonadaceae, Rikenellaceae*, unclassified *Clostridiales*, and others (Figure 3A). The percentile abundances of *Elusimicrobiaceae* (including “others”) were significantly lower in the DSS group (0.00%) than in the CON group (0.06%) using ANCOM statistics. In addition, the observed OTUs (representing alpha diversity) were lower in the DSS group than in the CON group (Figure 3B). A 3D principal coordinates analysis (PCoA) plot constructed from the unweighted UniFrac matrix of the cecal microbiota (representing beta diversity) identified a significant difference between the CON and DSS groups (Figure 3C, *p* = 0.0433, q = 0.0433).

### Egg Laying Rate and Egg Weight

**Figure 4:**
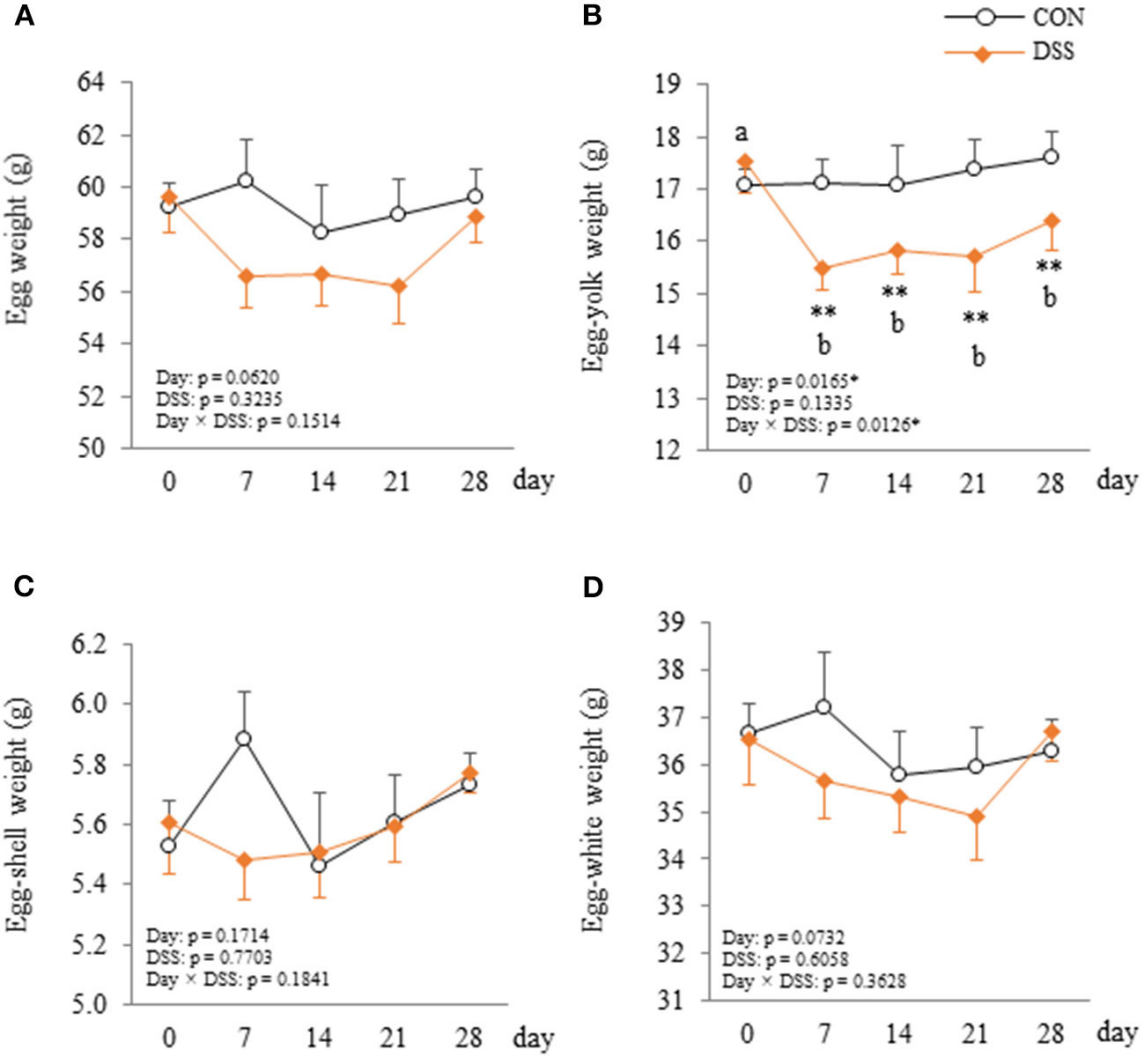
Changes in the egg **(A)**, egg-yolk **(B)**, egg-shell **(C)**, and egg-white **(D)** weight in hens administered dextran sodium sulphate (DSS group) or water (CON group). Open circle plots represent the CON group, and the orange rhombus plots represent the DSS group. Values are the mean ± SEM (CON and DSS, *n* = 8 and 7). Asterisks (**) indicate significant differences between the CON and DSS groups at the same time point (*P* < 0.01). Lower case letters (^a,b^) indicate significant differences between the time points (*P* < 0.05).

Egg laying rate during the experimental period in the DSS group (92.3 ± 3.7%) was significantly lower than that in the CON group (98.7 ± 0.6%) (*P* < 0.05). Egg-yolk weight was significantly lower in the DSS group than in the CON group at days 7, 14, 21, and 28, and the yolk weight in DSS group decreased from day 7 to 28 when compared with the first day (Figure 4B). Whole egg, eggshell, and egg-white weights did not show any significant differences in the DSS group during the experimental period (Figures 4A,C,D).

### Liver Tissue Observation and VLDLy Production

**Figure 5:**
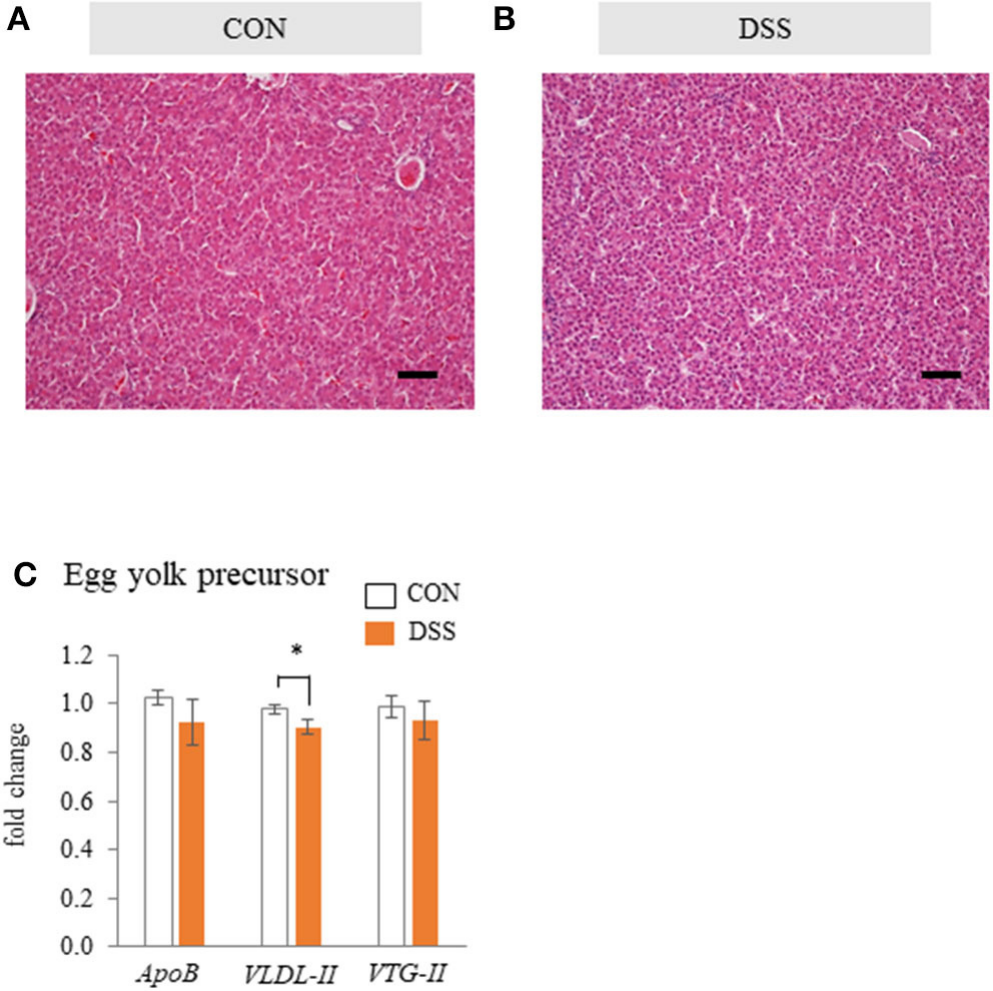
The effects of the oral administration of dextran sodium sulfate (DSS) on hepatic histology and function in hens. Micrographs of the liver tissue of hens treated with water [**(A)**; CON group] and DSS [**(B)**; DSS group]. HE staining. Scale bars = 50 μm. **(C)** Effects of the oral administration of DSS on the mRNA expression of egg yolk precursor related genes. Open bars represent the CON group, and orange filled bars represent the DSS group. Values demonstrate the fold change in the target gene expression when compared to a standard sample from the CON group of each segment (CON and DSS, *n* = 8 and 7). Target gene expression was normalized to the house-keeping gene *RPS17*. Asterisk (*) indicate significant difference between the control and DSS groups (*P* < 0.05).

The hepatic tissues of the CON and DSS groups were filled with hepatic cells and sinusoidal capillaries (Figures 5A,B). There was no observed difference between the CON and DSS groups in the liver tissue.

Gene expression levels of egg yolk precursor-related factors (*ApoB, VLDL-II*, and *VTG-II*) in the liver tissue are shown in Figure 5C. The expression of *VLDL-II* was significantly lower in the *DSS* group than in the CON group, but there was no significant difference in *ApoB* and *VTG-II*.

### Blood Parameter

**Figure 6:**
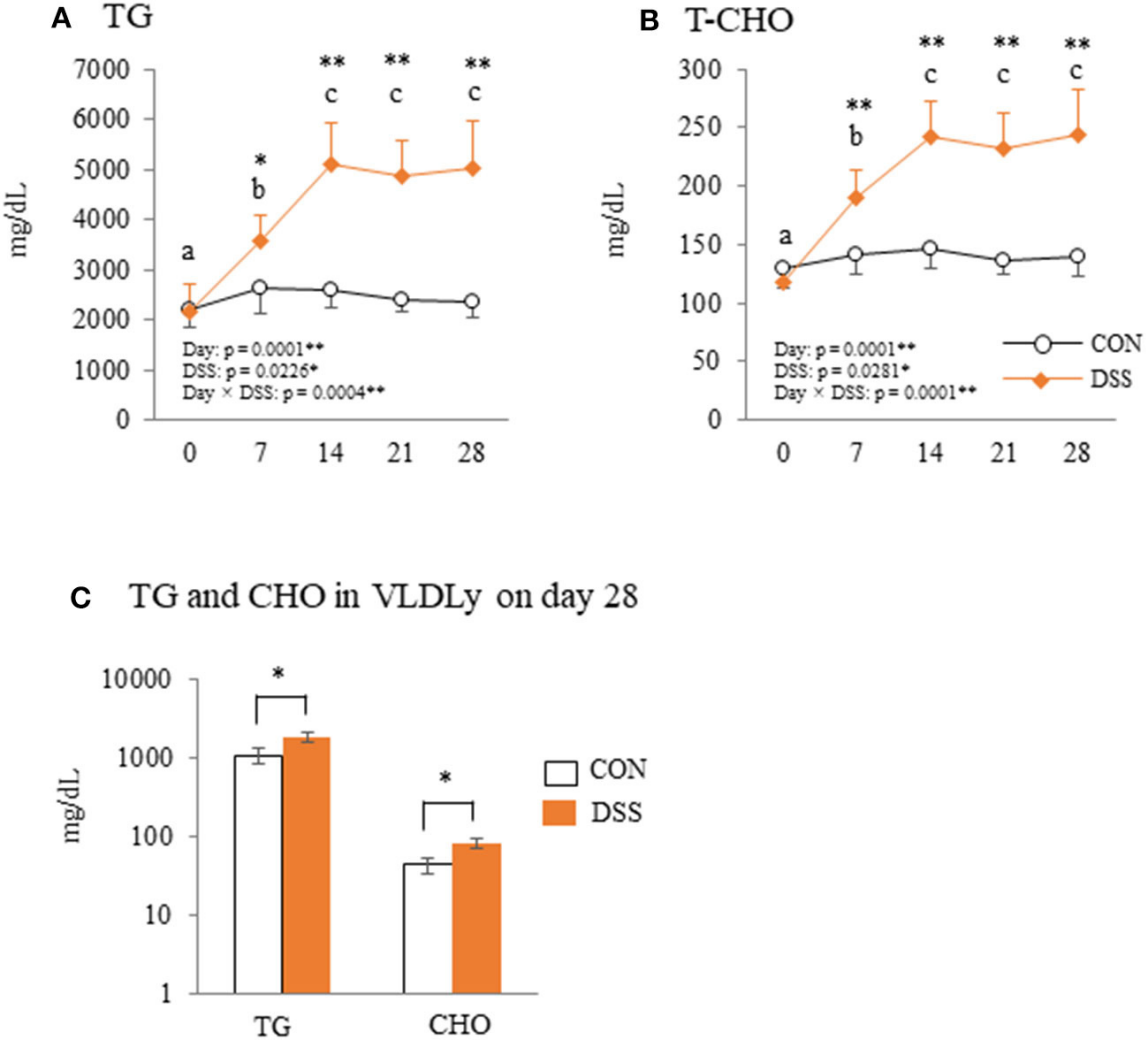
The plasma parameters of triglyceride (TG) and total cholesterol (T-CHO). Changes in plasma TG **(A)** and T-CHO **(B)** during the experimental period in hens administered dextran sodium sulfate (DSS group) or water (CON group). Open circle plots represent the control group, and the orange rhombus plots represent the DSS group. Values are the mean ± SEM (CON and DSS, *n* = 8 and 7). **(C)** The concentration of TG and cholesterol (CHO) in VLDLy (particle size with diameter of 25–44 nm) separated by high performance liquid chromatography (HPLC). Open circle plots represent the CON group, and the orange rhombus plots represent the DSS group. Asterisks (*, **) indicate significant differences between the control and DSS groups at the same time point (*P* < 0.05 and *P* < 0.01, respectively). Lower case letters (^a−c^) indicate significant differences between the time points (*P* < 0.05).

Blood plasma TG and T-CHO in the DSS group increased from day 7, peaked on day 14, then remained stable until day 28 (Figures 6A,B). Both TG and T-CHO were significantly higher in the DSS group than in the CON group from days 7 to 28. Both TG and CHO concentrations in the VLDLy particles in the blood plasma were higher in the DSS group than in the CON group (Figure 6C).

### Gene Expression of Tight Junction Proteins and Lipoprotein Receptors in the Follicular Granulosa Layer

**Figure 7:**
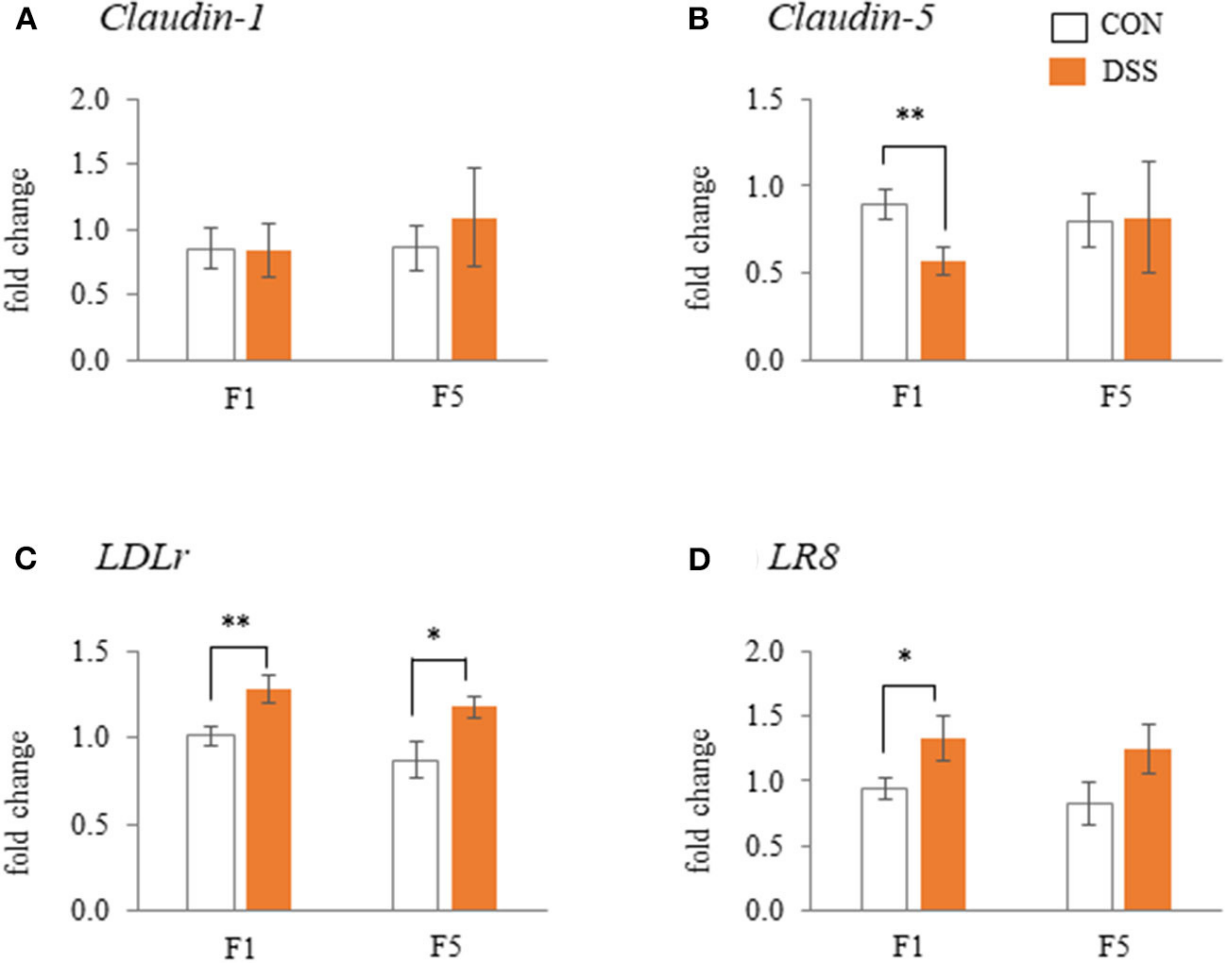
Effects of dextran sodium sulfate (DSS) on the mRNA expression of the tight-junction [**(A)** and **(B)**] and lipoprotein receptors [**(C)** and **(D)**] in the granulosa cells of F1 and F5 follicles. Open bars represent the CON group, and orange filled bars represent the DSS group. Values are the fold change in target gene expression when compared to a standard sample from the CON group of each segments (CON and DSS, *n* = 8 and 7). Target gene expression was normalized to the house-keeping gene *RPS17*. Asterisks (*, **) indicate significant differences between the water (control) and DSS-treated groups (*P* < 0.05 and *P* < 0.01, respectively).

The weight of the F1 follicle was 14.78 ± 0.79 g in CON group and 13.99 ± 0.35 g in DSS group. The F5 follicle weight was 1.78 ± 0.54 g in CON group and 2.12 ± 0.29 g in DSS group. There were no significant differences between the CON and DSS groups in both F1 and F5 follicles. Gene expression of *Claudin-5* in the F1 follicles was lower in the DSS group than in the CON group, but not in the F5 follicle (Figure 7B). DSS treatment did not affect *Claudin-1* gene expression in either F1 or F5 follicles (Figure 7A). The gene expression of *LDLr* in F1, F5, and *LR8* in F1 was significantly higher in the DSS group than in the CON group, but not in the *LR8* of the F5 follicle (Figures 7C,D).

### Gene Expression of Gonadotropin and *IL-1β* in the Anterior Pituitary Gland

**Figure 8:**
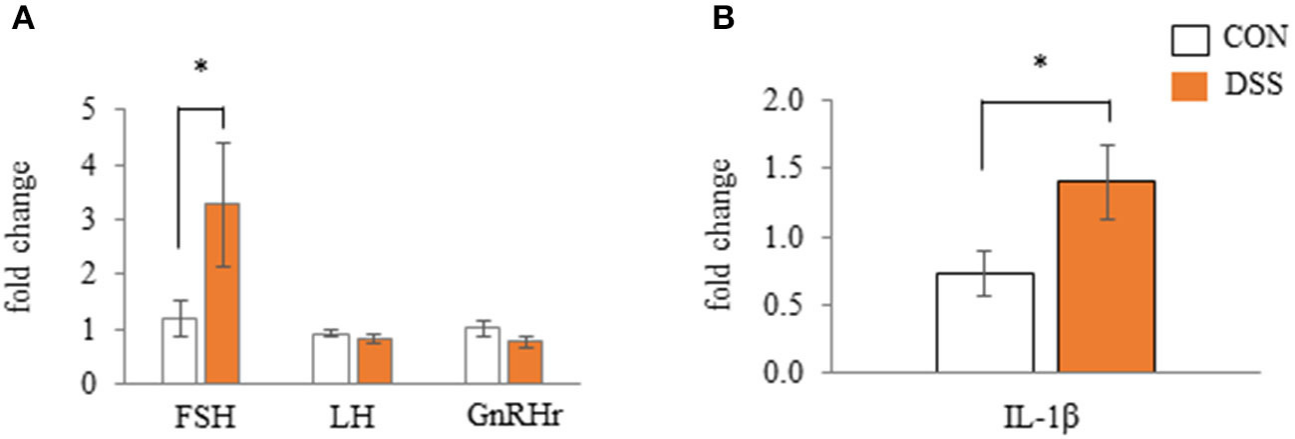
Effects of dextran sodium sulfate (DSS) on the mRNA expression of gonadotropin and receptor **(A)** and interleukin-1β **(B)** in the anterior pituitary gland. Values are the fold change in target gene expression when compared to a standard sample from the CON group of each segments (CON and DSS, *n* = 8 and 7). Target gene expression was normalized to the house-keeping gene *RPS17*. Asterisk (*) indicate significant differences between the water (control) and DSS-treated groups (*P* < 0.05).

The pituitary gland expressed the *FSH, LH*, and *GnRHr* gene. Only the *FSH* gene expression was significantly higher in the DSS group when compared to the CON group (Figure 8A). *IL-1*β (a pro-inflammatory cytokine) was higher in the DSS group than in the CON group (Figure 8B).

### Plasma Corticosterone and LBP Levels During the Experiment

**Figure 9:**
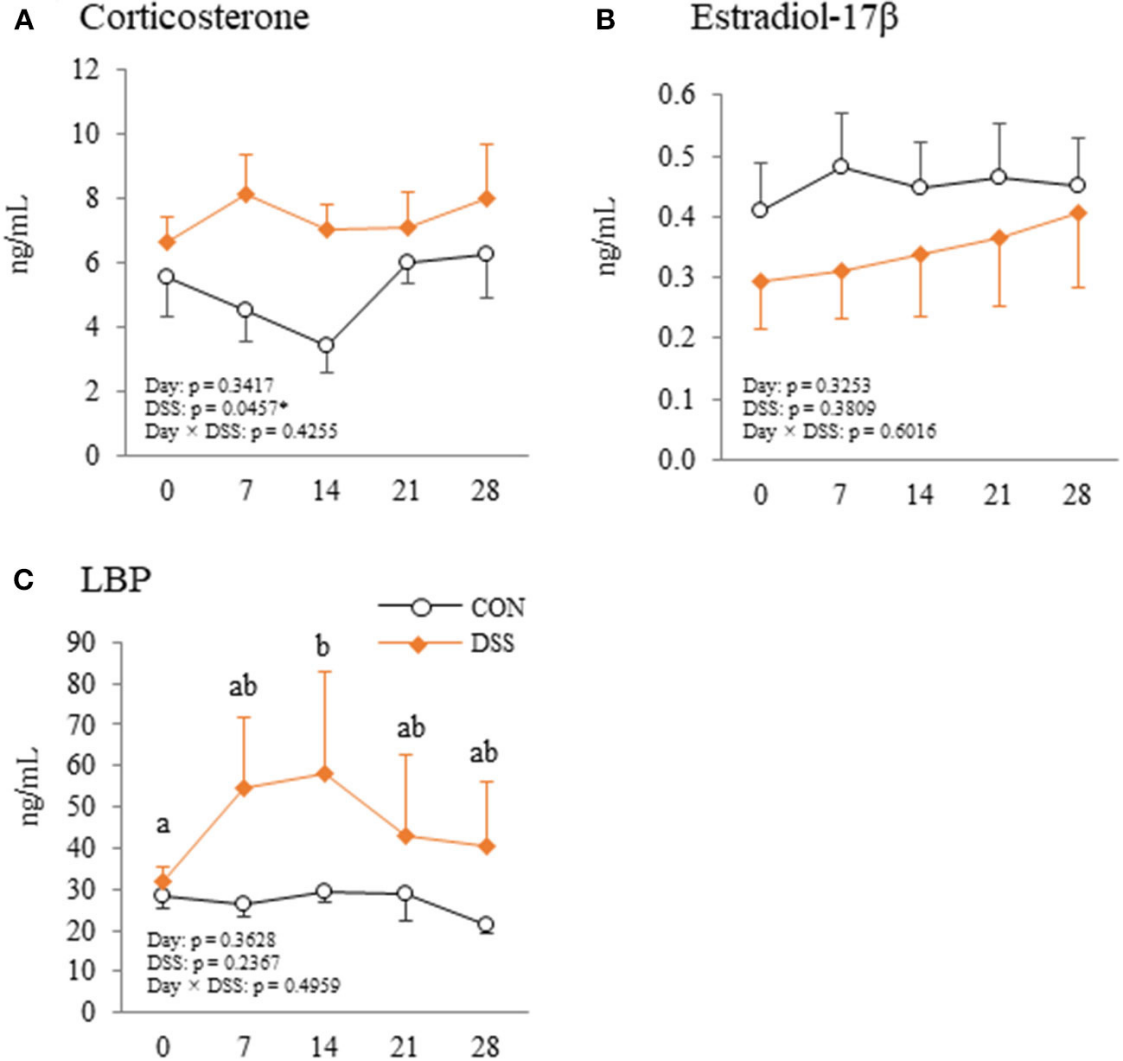
Changes in plasma corticosterone **(A)**, estradiol-17β **(B)**, and lipopolysaccharide binding protein (LBP) **(C)** during the experimental period in hens administered dextran sodium sulphate (DSS group) or water (CON group). Open circle plots represent the control group, and the orange rhombus plots represent the DSS group. Values are the mean ± SEM (CON and DSS, *n* = 8 and 7). Lower case letters (^a,b^) indicate significant differences between the time points (*P* < 0.05).

Corticosterone levels were higher in the DSS group than in the CON group, but the level did not vary with time in the DSS group (Figure 9A). Estradiol-17β concentrations did not vary with either DSS administration or time (Figure 9B). Plasma LBP concentration was significantly higher at day 14 than day 0 in DSS group (Figure 9C).

## Discussion

This report demonstrates the effects of the oral administration of a low dose of DSS on the intestinal environment and egg production in laying hens. The significant findings in this study identified that a lower concentration of orally administered DSS caused (1) a decrease in the ratio of villus height/crypt depth and *occludin* gene expression, *TGF-*β*4* expression, and diversity of cecal microbiota and an increase in *IL-6*, (2) a decrease in egg production and egg yolk weight, (3) no effect on the liver function that is related to yolk precursor production, and an increase in VLDLy in the blood plasma, (4) enhanced egg yolk precursor accumulation in the gene expression pattern in the follicular granulosa layer, and (5) an increase in *FSH* and *IL-1*β gene expression in the pituitary gland.

DSS has been used to induce intestinal inflammation. We previously orally administered 0.9 g DSS/kg BW to laying hens for five consecutive days. This caused histological disintegration of the mucosal epithelial tissue in the cecum, as well as an increased frequency of monocytes/macrophages, and increased expression of *IL-1*β, *IL-6, CXCLi2, IL-10*, and *TGF*β*-4* with a decrease in feed intake (Nii et al., 2020). The previous results suggested that the DSS administration caused heavy intestinal inflammation and damage to the intestinal mucosa. In the present study, we chose a lower DSS concentration than in our previous research. Oral administration of 0.225 g DSS/kg BW for 28 days increased *IL-6* gene expression in the cecum and decreased *TGF*β*-4* in the rectum (Figures 2B,C). In addition, DSS treatment decreased the ratio of villus/crypt in the cecum and rectum and *Occludin* gene expression in the cecum (Figures 1E,F). However, BW and feed intake did not change in the DSS group, and the specific signs of mucosal damage, such as infiltration of the blood cells and detachment of the epithelium, were not observed in any of the intestinal segments in the DSS group (Figures 1A–D). It has been reported that the ratio of villus/crypt in the intestinal mucosa decreases under inflammatory conditions, for example, during an *Eimeria* infection (Li et al., 2018). The decrease in the villus/crypt ratio has been widely acknowledged as a biomarker of poor intestinal health (Ducatelle et al., 2018). Therefore, the oral administration of a lower concentration of DSS in this study caused poor intestinal morphology without severe symptoms. Furthermore, DSS treatment decreased the alpha-diversity in the cecal contents. The beta-diversity was different between the DSS group and the CON group (Figures 3B,C). The presence of *Elusimicrobaceae* was eliminated by DSS administration in the present study. The character of *Elusimicrobaceae* remains unknown, but its population increases with probiotic treatment in the human intestine (Nagpal et al., 2018). It is known that DSS-induced colitis causes dysbiosis, including decreased microbiota diversity (Munyaka et al., 2016; Liu et al., 2020). Our results suggest that low dose DSS treatment may disrupt the intestinal environment, causing poor intestinal morphology and decreased microbiome diversity in the lower parts of the intestinal tract.

Direct-fed microbiotics (containing *Lactobacillus acidophilus, Lactobacillus casei, Enterococcus faecium*, and *Bifidobacterium thermophium*) are known to balance microorganisms and increase egg size in laying hens (Davis and Anderson, 2002). This report suggests that the intestinal environment may affect egg size. DSS treatment slightly reduced the ratio of egg production and clearly decreased egg yolk weight in this study (Figure 4B). However, the weight of the whole-egg, eggshell, and egg white in the DSS group did not change during the experiment (Figures 4A,C,D). Our results suggest that DSS induced disruption in the intestinal environment and suppressed egg yolk accumulation into the ovarian follicle, but did not have a strong effect on oviduct function, such as egg white and eggshell formation.

Nutritional conditions are important factors that affect egg quality. In general, intestinal inflammation causes malnutrition through a decrease in feed intake, digestion, and absorption functions (Peuhkuri et al., 2010). Malnutrition caused by feed restriction (60–80% decrease compared with normal feed intake) decreased not only the whole-egg weight, but also egg-shell thickness with a decrease in plasma TG in laying hens in our previous study (Nii et al., 2020). In addition, the plasma TG level of molting hens (caused by feed removal) was significantly decreased during the molting phase (Meng et al., 2013). In the present study, feed intake and BW did not decrease with slight intestinal inflammation. In addition, plasma TG levels increased from day 7 to 28 in the DSS group (Figure 6A). The dynamics of TG concentration in this study are completely different to malnutrition caused by feed restrictions that show low blood TG levels (Landers et al., 2008). Furthermore, in general, malnourished hens produce thin eggshell eggs. The eggshell weight did not change during the experimental period in the DSS group in this study (Figure 4C). Therefore, it is assumed that the nutrient condition is not the sole factor suppressing egg yolk size in the DSS group in this study.

VLDL and VTG are the main components of egg yolk precursors. Specific VLDLs, with a particle size of 25–44 nm, are called “VLDLy” that accumulates in developing egg yolk (Yang et al., 2013). VLDLy is formed with 23 units of VLDL-II (known as ApoVLDL-II) and 1 unit of apolipoprotein (apo) B100 (ApoB), and it is produced in the liver (Bujo et al., 1997; Walzem et al., 1999). We previously reported that rapid severe intestinal inflammation caused by a higher concentration of DSS induced liver tissue inflammation, and the gene expression of lipid synthesis and egg yolk precursor production were significantly decreased in the liver (Nii et al., 2020). Therefore, we hypothesized that the decrease in egg yolk size was also caused by dysfunction of the production of the egg yolk precursor in the liver in the present study. However, liver tissue did not show any apparent damage in the DSS group (Figures 5A,B). In addition, the gene expression of *ApoB* and *VTG-II* was not changed in the DSS group. The *VLDL-II* was decreased in DSS group, but the value did not show a noticeable difference between the CON and DSS groups (Figure 5C). In contrast, TG and CHO levels in the VLDLy fraction directly reflecting the plasma VLDLy quantity was higher in the DSS group than in the CON group (Figure 6C). High concentrations of plasma TG and T-CHO in the DSS group may reflect a high quantity of VLDLy in the blood in the DSS group (Figures 6A,B). These results suggest that the liver maintained the function for yolk precursor production, and a sufficient amount of VLDLy was supplied from the liver into the blood. Nonetheless, VLDLy may not be utilized for follicle development in the ovary.

As mentioned above, we expected the yolk precursor uptake from blood to the follicle might be disrupted by DSS administration. The gene expression of *Clauin-5* was decreased. The *LDLr* in F1 and F5 follicles, and *LR8* in F1 follicles were increased in the granulosa layer of the DSS group (Figures 7B–D). During follicular growth, the egg yolk precursor uptake in ovarian follicles is enhanced by an increase in lipoprotein receptor expression and a weakening in the tight junction connection (Schuster et al., 2004; Stephens and Johnson, 2012; Rosewell et al., 2013). Hence, the present results of gene expression of *Claudin-5* and lipoprotein receptors suggested that ovarian follicles in the DSS group more actively took up yolk precursor than in the CON group, and the activity may be higher in the F1 than in the F5 follicles. LDLr protein expression in follicular granulosa cells did not change among F1, F2, and F3 follicles (Hummel et al., 2003). *LDLr* and *LR8* gene expression did not change during yellow follicle growth in White Leghorn laying hens (Seol et al., 2007). In addition, the weight of F1 and F5 follicles did not show significant differences between the CON and DSS groups in the present study. This result suggests that the difference in *Claudin-5, LDLr*, and *LR8* gene expression in the DSS group did not depend on follicular size. Moreover, *FSH* gene expression in the pituitary gland was significantly higher in the DSS group than in the CON group in the present study (Figure 8A). FSH secreted from the anterior pituitary gland enhances follicle growth in the ovary. Therefore, the change in *Claudin-5, LDLr*, and *LR8* gene expression in the granulosa layer may reflect the activation of follicle growth with an increase in FSH expression in the pituitary gland. Thus, it seems that ovarian follicles in the DSS group are activated by yolk precursor uptake through changes in the expression of *Claudin-5* and lipoprotein receptors. Conversely, the actual yolk precursor uptake was suppressed. Further research is necessary to determine why follicle growth was suppressed by DSS induced disruption of the intestinal environment.

*IL-1*β, a pro-inflammatory cytokine, increased with stress conditions in the pituitary glands of mice (Nguyen et al., 2000). Intracerebroventricular injection of IL-1β increases plasma corticosterone and adrenocorticotropic hormone (ACTH) in rats (Brown et al., 1991). High plasma levels of corticosterone mediated by corticosterone pellet transplantation decreased the egg yolk mass in White Leghorn hens (Henriksen et al., 2011). These reports assumed that secreted IL-1β in the pituitary gland may increase blood corticosterone levels, and that corticosterone decreases egg yolk size in laying hens. In the present study, gene expression of *IL-1*β in the pituitary gland was significantly higher in the DSS group than in the CON group (Figure 8B), but the plasma corticosterone level did not change during the experimental period in both the CON and DSS groups (Figure 9A). Meanwhile, IL-1β injection into the brain caused a total disruption of the estrous cycle with a decrease in mRNA expression of LH and FSH in the pituitary gland as well as their plasma levels in rats (Rivest et al., 1993). However, the mRNA expression of FSH increased, and LH was not changed in the DSS group when compared to the CON group (Figure 8A), and the plasma estradiol-17β concentration in the DSS group did not change over time in the present study (Figure 9B). Therefore, it is unknown whether the increase in IL-1β in the pituitary gland affects the decrease in egg yolk size in the present study.

We previously reported that severe intestinal inflammation caused by DSS may lead to an influx of enteric gram-negative bacteria or LPS from the cecum to the liver through the portal vein (Nii et al., 2020). In addition, destruction of the intestinal mucosal barrier led to the invasion of enteric bacteria into the lamina propria, followed by an influx of bacteria and/or endotoxin to other organs through the blood stream (Jahnel et al., 2009; Qin et al., 2018). LPS stimulation increased the apoptosis level of follicular ovarian granulosa cells in cows (Wang et al., 2020). This report suggests that LPS contaminated blood may disrupt normal follicular growth. In the present study, DSS orally administration caused slight mucosal inflammation in the large intestine and decreased *occludin* gene expression in the cecum (Figures 1F, 2). In addition, the plasma LBP level, which indirectly indicates plasma LPS level, was increased in the DSS group (Figure 9C). Therefore, the endotoxin originating from the intestine by the bloodstream is likely to be one of the factors that cause dysfunction in egg yolk precursor accumulation in ovarian follicles.

The research showed the importance of the intestinal environment for poultry egg production. Specifically, weakened intestinal mucosal barrier function may result in an increase in endotoxin influx into the blood stream derived from intestinal contents, and it may inhibit ovarian follicular growth and causes decrease in egg-yolk size. Therefore, it is expected that an enhancement of intestinal mucosal barrier function improves egg-yolk size. Some feed additive such as probiotics and organic acid was reported to enhance intestinal mucosal barrier function (Wang et al., 2018; Yang et al., 2019). Thus, these materials are expected to contribute to the improvement of egg-yolk size in poultry farming. In addition, the DSS method used in this study is useful for the studies on poor intestinal environment model in hens. The method is expected to be used for screening of some materials which have ameliorating effects on the intestinal environment and egg production in laying hens.

Therefore, the lower concentration of DSS administered orally caused a slight disruption in the intestinal environment. This disruption included poor intestinal morphology and decreased the cecal microbiome diversity. The change in the intestinal environment decreased the egg yolk size without decreasing the VLDLy supply from the liver. The decrease in egg yolk size is likely to be caused by dysfunctional egg-yolk precursor uptake in the ovarian follicles in association with increase in the influx of LPS. In conclusion, the oral administration of a lower dose of DSS is useful method to causes slight disruption of intestinal environment, and the disrupted intestinal condition decreases egg yolk size through disfunction of ovarian follicle.

## Data Availability Statement

The datasets generated for this study can be found in online repositories. The names of the repository/repositories and accession number(s) can be found at: https://www.ddbj.nig.ac.jp/, DRA010795.

## Ethics Statement

The animal study was reviewed and approved by Hiroshima University Animal Research Committee.

## Author Contributions

TN: conceived, designed, performed, and analyzed the experiments. NI: ELISA analysis of corticosterone and estradiol-17β. TB and YY: critical discussion and reviewing of the manuscript. All authors contributed to the article and approved the submitted version.

## Conflict of Interest

The authors declare that the research was conducted in the absence of any commercial or financial relationships that could be construed as a potential conflict of interest.
